# 

**Published:** 1891-04-25

**Authors:** 


PRICE ONE PENNY.

No. 239, Vol. X.—April 25th, 1891.

[Registered at the General Post Office as a Newspaper for transmission in the United Kingdom and Abroad.]

WITH EXTRA NURSING SUPPLEMENT.

Per Annum, 6s. 6d.; post free.

Monthly Parts, 6d.; post free 8d.

Ditto per Annum, 8s., post free.

### THE HOSPITAL

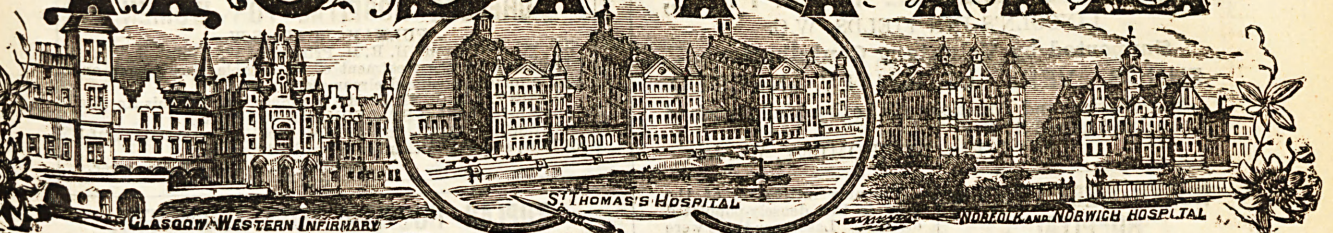

A WEEKLY JOURNAL OF

### Science, Medicine, Nursing and Philanthropy.

SATURDAY, APRIL 25th, 1891.

**Prescriptions and Their Owners** - 37

**Passing Topics.**—Voting Charities - 38

**The Doctor's Armchair.**—A Clerical Publican - 39

**The History and Capabilities of Herbal Simples**—By W. T. Fernie, M D. XXX.—The Dock - 40

**The Microscope in Medicine**—By Frank J. Wethered, M.D., XII.—The Examination of Urinary Deposits (*continued*) - 41

**Everybody's Page** - 41

**Notes and News** - 42

**General Charities** - 43

**Annotations**—Logical Women—The Radcliffe Infirmary, Oxford—The Tomb of Aristotle - 44

**Scraps and Gleanings** - 43

**The Practitioner's Mirror and Retrospect:—**

Medicine.—Hepatic Colic - 45

Surgery.—Ingleby Lectures: Lecture III. - 45

Therapeutics.—On the Pharmacology of Iron - 46

New Drugs and Appliances.—Listerine—New Ragocyi Effervescing Saline Ferruginous Water, 47; Peterman's Cockroach and Beetle Food (Non-Poisonous)—Claxton's Patent Ear Cap - 48

**The Lords' Committee** - 48

**The Student of Medicine.**—The Students' Bookshelf—Appointments—Vacancies—Scholarships and Prizes—Presentation—Pass Lists

EXTRA SUPPLEMENT—THE NURSING MIRROR.

**En Passant.**—Influenza Again—Work in Wales—Religion at Norwich—A Serious Warning—Another Nursing Chart—Short Items—Presentations at Burnley—Norwich Hospital - xix

**Lectures on Surgical Ward Work and Nursing.**—By Alex. Miles, M.B. Edin., C.M., F.R.C.S.E.—Lecture XXI.—Rest - xx

**The Eastern Hospital Scandal** - xxi

**The Sarah Acland Home for Nurses, Oxford** - xxii

**Everybody's Opinion.**—Flowers for Hospitals—A Pro.'s Grievances - xxiii

**The Registration of Nurses** - xxiii

**Presentations** - xxiii

**Notes and Queries** - xxiv

**Off Duty.**—Pat. - xxiv

**Appointment—Amusements and Relaxation** - xxiv

